# Broadly Sarbecovirus-Neutralizing Antibodies Induced by Ancestral SARS-CoV-2 Infection

**DOI:** 10.3390/v17101285

**Published:** 2025-09-23

**Authors:** Yiwei Zhang, Zhen Zhang, Feiyang Yu, Xianying Chen, Shangyu Yang, Jingyi Lin, Genmao Liu, Xinyang Liu, Ming Guo, Yu Chen, Ke Lan, Haiyan Zhao

**Affiliations:** 1 State Key Laboratory of Virology and Biosafety, College of Life Sciences, Wuhan University, Wuhan 430072, China; 2025102040018@whu.edu.cn (Y.Z.); yufeiy@whu.edu.cn (F.Y.); 2021202040060@whu.edu.cn (S.Y.); 2024202040040@whu.edu.cn (J.L.); 2024202040047@whu.edu.cn (G.L.); 2020302042013@whu.edu.cn (X.L.); guoming@whu.edu.cn (M.G.); chenyu@whu.edu.cn (Y.C.); 2 Animal Biosafety Level-III Laboratory/Institute for Vaccine Research, Wuhan University, Wuhan 430071, China; zhangzhen@whu.edu.cn (Z.Z.); chenxianying@whu.edu.cn (X.C.)

**Keywords:** SARS-CoV-2, SARS-CoV-1, sarbecoviruses, receptor-binding domain, neutralizing antibodies, inhibitory mechanisms

## Abstract

The COVID-19 pandemic, driven by SARS-CoV-2, continues to challenge global health due to emerging variants and the potential risk posed by related sarbecoviruses. Neutralizing antibodies targeting the spike (S) glycoprotein, particularly the receptor-binding domain (RBD), play a crucial role in viral neutralization and vaccine design. Although broadly neutralizing anti-RBD antibodies have been identified, the nature of cross-reactive humoral responses induced by natural infection with ancestral SARS-CoV-2 strains remains incompletely understood. Here, we isolated 105 S-specific monoclonal antibodies (mAbs) from individuals recovered from prototype SARS-CoV-2 infection. Of these, 30 mAbs cross-recognized SARS-CoV-1, including 25 RBD-directed mAbs, of which 12 displayed cross-neutralizing activity against both viruses. Among them, mAb 12C2 potently neutralized SARS-CoV-1 and multiple SARS-CoV-2 variants, likely through mechanisms that include inhibition of membrane fusion and potential destabilization of the S trimer. Cryo-electron microscopy revealed that 12C2 engages the outer face of the RBD, overlapping with the epitope recognized by the broadly neutralizing antibody S309 derived from SARS-CoV-1 convalescent. Collectively, these findings demonstrate that ancestral SARS-CoV-2 infection can elicit robust cross-neutralizing antibody responses and provide valuable insights for the design of broadly protective antibodies and vaccines.

## 1. Introduction

The coronavirus disease 2019 (COVID-19) pandemic, caused by severe acute respiratory syndrome coronavirus 2 (SARS-CoV-2), has profoundly impacted global health and economies [1,2,3]. Despite the availability of multiple vaccines and therapeutic agents, the continuous emergence of SARS-CoV-2 variants poses significant challenges to long-term pandemic control [4,5,6]. Moreover, the potential threat of severe acute respiratory syndrome coronavirus (SARS-CoV-1) [7], which, like SARS-CoV-2, belongs to the subgenus *Sarbecovirus* within the genus *Betacoronavirus* [8], underscores the urgent need for the development of effective therapies and vaccines to address potential sarbecovirus pandemics.

Neutralizing antibodies are a crucial component of the host’s antiviral defense and have proven valuable in the clinical prophylaxis and therapy of COVID-19. Protective epitopes for coronaviruses are primarily concentrated on the spike (S) glycoprotein, which plays a critical role in virus entry by interacting with the host cell receptor, angiotensin-converting enzyme 2 (ACE2) [9,10]. The S protein is a class I fusion protein that appears as a trimer in both pre- and post-fusion states [11,12]. Structural analyses have revealed that the S protein comprises the receptor-binding S1 subunit (containing the receptor-binding domain [RBD] and N-terminal domain [NTD]) and the S2 subunit, which is responsible for viral fusion with the host cell membrane [11]. Neutralizing antibodies targeting all three structural domains, RBD, NTD, and S2, have been identified, exhibiting diverse mechanisms, including receptor binding blockade, stabilization of the spike pre-fusion conformation, cross-linking of viral proteins, inhibition of membrane fusion, and facilitation of immune clearance [13,14,15,16].

While NTD- and S2-targeting antibodies exhibit broad-spectrum recognition capabilities, their neutralization potency is generally limited [17,18,19,20,21]. In contrast, RBD-targeting antibodies often demonstrate potent inhibitory activity and present the best prospects for application. Notably, several broadly neutralizing antibodies against the sarbecovirus RBD, particularly those cross-neutralizing both SARS-CoV-1 and SARS-CoV-2, have been reported [22,23,24,25,26]. Based on their binding epitopes, anti-RBD antibodies are classified into eight groups, with the majority of cross-neutralizing mAbs belonging to the RBD-2, RBD-5, RBD-6, RBD-7, and RBD-8 communities [27,28,29]. In addition, some broadly reactive RBD antibodies are nanobodies [30,31,32]. While repeated immunization can induce potent cross-neutralizing antibodies, those generated by natural infection often better reflect the humoral immune response against viral infection.

Most human cross-neutralizing anti-RBD antibodies arise from SARS-CoV-1 convalescents, individuals who have received multiple SARS-CoV-2 vaccinations, those infected with SARS-CoV-2 variants, or from a combination of vaccination and natural infection [22,24,33,34,35]. For instance, cross-neutralizing monoclonal antibodies (mAbs) such as S309 and CR3022 were isolated from SARS-CoV-1 convalescents [22,24]; PW5-5 and PW5-535 from a SARS-CoV-2-vaccinated individual [33]; SA55 from a SARS-CoV-2-vaccinated SARS-CoV-1 convalescent [34]; and S2V29 from an Omicron-infected SARS-CoV-2 vaccinee [23]. However, the characteristics and magnitude of the cross-reactive antibody response between SARS-CoV-1 and SARS-CoV-2, particularly in convalescents infected with ancestral SARS-CoV-2 strains, remain poorly understood.

In this study, we isolated 105 spike-recognizing mAbs from convalescent donors infected with the prototype SARS-CoV-2 strain (Wuhan-Hu-1) using the S1 protein as bait. Among these, 30 mAbs cross-recognized SARS-CoV-1, including 25 that targeted the RBD, with 12 exhibiting cross-neutralizing activity against both SARS-CoV-1 and SARS-CoV-2. Notably, mAb 12C2 demonstrated the most potent neutralization against SARS-CoV-1 and multiple early SARS-CoV-2 variants, including D614G, Alpha, Beta, Gamma, and Delta. Mechanistic studies revealed that 12C2 likely inhibits viral infection through multiple mechanisms, including fusion inhibition and disruption of the Spike trimer. Cryo-electron microscopy (cryo-EM) analysis of the Spike-12C2 complex showed that 12C2 binds the outer face of the RBD, a region also targeted by S309 isolated from a SARS-CoV-1 convalescent. These findings indicate that infection with ancestral SARS-CoV-2 elicits a robust cross-reactive humoral immune response against SARS-CoV-1 and provides valuable insights for the development of next-generation vaccines targeting diverse sarbecoviruses.

## 2. Materials and Methods

### 2.1. Viruses and Cells

All SARS-CoV-2 authentic viruses were provided by the Animal Biosafety Level-III Laboratory (ABSL-3) of Wuhan University. BHK-hACE2 cells were kindly provided by Dr. Huan Yan from Wuhan University. Vero E6 and 293T cells were previously preserved by our laboratory and maintained in Dulbecco’s Modified Eagle’s Medium (DMEM; Monad, Wuhan, China) supplemented with 8% or 10% heat-inactivated fetal bovine serum (FBS; ExCell Bio, Suzhou, Jiangsu, China).

### 2.2. Plasmids

Plasmids encoding the spike proteins of SARS-CoV-1, MERS-CoV, RaTG13, and SARS-CoV-2 variants (D614G, Alpha, Beta, Gamma, Delta, Omicron BA.5 and XBB) were kindly provided by Dr. Huan Yan from Wuhan University. The expression plasmid for the SARS-CoV-2 prefusion spike ectodomain trimer was generated as previously described [36,37]. For other human coronavirus prefusion trimer expression vectors, modifications were introduced into DNA fragments encoding the spike ectodomains: SARS-CoV-1 (GenBank accession: AAP13567.1, residues 14–1193) included two proline substitutions at residues 968 and 969, while MERS-CoV (GenBank accession: YP_009047204.1, residues 19–1294, S2P) contained a ‘ASVG’ furin cleavage site mutation at residues 748–751 and two proline substitutions at residues 1060 and 1061. The SARS-CoV-1 S2P and MERS-CoV S2P fragments were individually cloned into the pCAGGS vector downstream of a CD5 signal peptide and terminated with a T4 fibritin trimerization motif, an HRV-3C cleavage site, and a Strep-tag II.

To express the SARS-CoV-2 receptor-binding domain (RBD), the RBD sequence (GenBank accession: YP_009724390.1, residues 319–541) was cloned into pCAGGS vector with an N-terminal CD5 signal peptide, a C-terminal HRV-3C cleavage site, and a 6 × His tag. For the SARS-CoV-2 S2 construct, residues 697–1208 of the spike protein were used, incorporating proline substitutions at residues 817, 892, 899, 942, 986, and 987, along with an N-terminal CD5 signal peptide, a C-terminal HRV-3C protease recognition site, and a Strep-tag II, all cloned into pCAGGS vector. All plasmid constructs were validated by Sanger sequencing.

### 2.3. Protein Expression and Purification

Plasmids were transfected into Expi293 cells using polyethyleneimine (PEI; Polysciences, Warrington, PA, USA) at a DNA:PEI ratio of 1:1.5 or Carptrans (OPM Biosciences, Shanghai, China) at 1:4. Culture supernatants were harvested 108 h post-transfection by centrifugation and filtered through a 0.45 μm membrane. His-tagged proteins were purified from the supernatants using Ni-Charged Resin (GenScript, Nanjing, China), while Strep-tagged proteins were purified using STarm Streptactin Beads 4FF (Smart-Lifesciences, Changzhou, China). Further purification was performed by size exclusion chromatography on a Superdex 200 Increase column or Superose 6 10/300 GL column (Cytiva, Uppsala, Sweden). Purified proteins were concentrated, flash-frozen, and stored at −80 °C.

### 2.4. S1-Specific Memory B Cell Sorting

Peripheral blood mononuclear cells (PBMCs) were isolated from COVID-19 convalescent blood samples using Lymphocyte Separation Tubes for Human Peripheral Blood (DAKEWE, Shenzhen, China) according to the manufacturer’s instructions. PBMCs were stained with biotinylated SARS-CoV-2 S1 protein, followed by fluorescent staining with FVS-780, anti-CD3, anti-CD4, anti-CD8, anti-CD19, anti-IgD, anti-CD20, and Streptavidin-APC. Antigen-specific plasma B cells (CD19^+^CD3/4/8^−^IgD^−^S1^+^) were sorted into 96-well plates with one cell per well using FACS Aria ΙΙΙ flow cytometer (BD Biosciences, San Jose, CA, USA) and stored at −80 °C after flash freezing by dry ice.

### 2.5. Monoclonal Antibody (mAb) Generation

The variable region sequences of paired mAbs were determined and amplified using a previously established protocol. Briefly, the V_H_ and V_L_ segments of the antibody were obtained from the single B cells via reverse transcription and nested-PCR, then individually cloned into mammalian expression vectors containing human IgG1 constant regions of heavy and light chains, respectively. For Fab expression, the V_H_ segments were also cloned into a modified heavy chain expressing vector terminated with a 6 × His tag following the CH1 constant region. Expression and purification of mAbs and Fabs were performed using rProtein A Beads (Smart Lifesciences, Changzhou, China) or Ni-Charged Resin (GenScript, Nanjing, China) as described above.

### 2.6. Enzyme-Linked Immunosorbent Assay (ELISA)

96-well ELISA plates (Corning, Corning, NY, USA) were coated with antigen (3 µg/mL, 50 µL/well) overnight at 4 °C or for 2 h at 37 °C. Following three washes with PBS containing 0.05% Tween-20 (PBS-T), the plates were blocked with 1% bovine serum albumin (BSA) in PBS-T for 2 h at 37 °C. After another three PBS-T washes, test antibodies diluted in 1% BSA/PBS-T were added and incubated for 2 h at 37 °C. After washing, HRP conjugated goat anti-human IgG (1:20,000 dilution; ABclonal, Wuhan, China) was added and incubated for 1 h at 37 °C. Plates were washed three times with PBS-T, followed by addition of one component TMB chromogen solution (NCM Biotech, Suzhou, China) and incubation for 15 min (protected from light). The reaction was stopped by adding 1 M HCl (50 µL/well), and the absorbance was measured at 450 nm (OD_450_) using Varioskan LUX microplate reader (Thermo Fisher, Waltham, MA, USA).

### 2.7. Flow Cytometry

293T cells were transfected to overexpress coronavirus spike proteins using GeneTwin (Biomed, Beijing, China). 48 h post-transfection, cells were harvested by centrifugation, fixed with 4% paraformaldehyde (PFA), and resuspended in PBS containing 1% BSA (FACS buffer). Cells were incubated on ice with test monoclonal antibodies (1 µg/mL in FACS buffer) for 1 h, washed twice, then stained with Alexa Fluor 488-conjugated anti-human IgG antibody (1:1000 dilution; Invitrogen, Carlsbad, CA, USA) for 30 min. After two additional washes with FACS buffer, samples were analyzed on a CytoFLEX S flow cytometer (Beckman Coulter, Brea, CA, USA), and data were processed using FlowJo v10 software.

### 2.8. Pseudovirus Production and Infectivity

SARS-CoV-1 (GenBank: AAP13567.1), SARS-CoV-2 (GenBank: BEI32265.1), and MERS-CoV (GenBank: YP_009047204.1) spike proteins were pseudotyped onto VSVΔG-eGFP particles. Briefly, 293T cells were transiently transfected with plasmids encoding full-length spike proteins using GeneTwin (Biomed, Beijing, China) and cultured in DMEM supplemented with 10% FBS (ExCell Bio). At 24 h post-transfection, cells were infected with VSVΔG-eGFP in DMEM containing 4% FBS for 6 h. Cells were then washed three times with PBS and incubated in DMEM with 4% FBS plus anti-VSV-G monoclonal antibody I1 (1 μg/mL) for an additional 24 h. Pseudovirus-containing supernatants were clarified by centrifugation, aliquoted, and stored at −80 °C. Infectivity of SARS-CoV-1 and SARS-CoV-2 pseudoviruses was assessed using serial dilution infection assays on BHK21-hACE2 cells, whereas MERS pseudovirus infectivity was tested on Vero E6 cells.

### 2.9. Pseudovirus Neutralization Assay

For the pseudovirus neutralization assay, antibodies were serially diluted four-fold starting from 50 µg/mL in 96-well plates. Then, 50 µL of diluted pseudovirus was added into 50 µL serial dilutions of mAb and the mixtures were incubated for 1 h at 37 °C. The pseudovirus-mAb mixtures were added to BHK21-hACE2 or Vero E6 cells at approximately 90% confluence which were seeded in 96-well plates the day before infection. After 24 h, cells were fixed with 4% PFA (Biosharp, Hefei, China) for 15 min at room temperature. Green fluorescent spots (GFP) were quantified using a ImmunoSpot S6 Ultra M2 Analyzer (Cellular Technology, Shaker Heights, OH, USA). Half-maximal inhibitory concentration (IC_50_) values were calculated by fitting nonlinear regression curves using GraphPad Prism v9.5.0 software.

### 2.10. Authentic Virus Neutralization

Focus reduction neutralization tests (FRNT) were used to determine neutralization activity of mAbs against authentic SARS-CoV-2 viruses. Vero E6 cells were seeded in 96-well plates (2 × 10^4^ cells/well in DMEM with 10% FBS) and incubated overnight at 37 °C until reaching 90–100% confluence. Then, serial four-fold dilutions of antibodies were mixed with virus suspension (2400 PFU/mL) and incubated at 37 °C for 1 h. The mixtures (100 µL per well) were added to Vero E6 cells and incubated for 1 h at 37 °C, then overlaid with 100 µL methylcellulose medium per well. After 24 h, cells were fixed with 4% PFA, permeabilized with 0.5% Triton X-100, and stained with SARS-CoV-2 N Protein Rabbit mAb (1:1000; ABclonal, Wuhan, China) followed by ABflo^®^ 488-conjugated Goat anti-Rabbit IgG (H + L) (1:100; ABclonal, Wuhan, China). Green fluorescent spots were counted and analyzed similarly to the pseudovirus neutralization assay.

### 2.11. Immunofluorescence Assay (IFA)

293T cells were transfected with full-length spike proteins of SARS-CoV-2 (GenBank accession: BEI32265.1), MERS-CoV (GenBank accession: YP_009047204.1), NL63 (GenBank accession: APF29071.1), HKU1 (GenBank accession: YP 173238.1), and the M segment of SFTSV (negative control). At 48 h post-transfection, cells were fixed with PFA, permeabilized with 0.5% Triton X-100 for 20 min, washed with PBS, and blocked with 2% BSA in PBS-T for 2 h. Cells were then incubated overnight at 4 °C with the indicated antibodies (10 μg/mL). After washing, cells were stained with Alexa Fluor™ 488 Goat anti-Human IgG (H + L) (1:1000; Invitrogen, Carlsbad, CA, USA) for 1 h at room temperature. Nuclei were counterstained with Hoechst (ThermoFisher, Waltham, MA, USA). Images were acquired using a SOPTOP ICX41 fluorescence microscope.

### 2.12. Cell Fusion and Inhibition Assay

A dual-functional split-reporter system, which includes the RL-DSP1-7 and RL-DSP8-11 expression vectors, was employed for the fusion inhibition assay as previously described [38,39]. Briefly, 293T cells co-transfected with plasmids encoding full-length SARS-CoV-2 spike and RL-DSP1-7 served as effector cells, while BHK21 cells stably expressing human angiotensin-converting enzyme 2 (BHK21-hACE2) transfected with plasmids encoding RL-DSP8-11 served as target cells. Spike glycoprotein-mediated cell-cell fusion was monitored via GFP fluorescence and luciferase activity.

To measure the inhibitory effect of antibodies on cell–cell fusion, 24 h post-transfection, antibodies 3E4, 12C2, and 12D1 were serially diluted in DMEM containing 10% FBS and added to the wells of a 96-well plate, with an initial concentration of 200 μg/mL and a 3-fold dilution gradient. Cells treated with medium without mAbs were used as controls. Effector cells were trypsinized and added to the wells at a density of 5 × 10^4^ cells per well, incubated at 37 °C for 1 h. Subsequently, target cells were added to the wells at a density of 5 × 10^4^ cells per well and incubated at 37 °C for an additional 3 h. The culture medium was then discarded and replaced with fresh medium containing 20 μM EnduRen live-cell substrate (Promega, Madison, WA, USA). After at least 2 h incubation, luciferase activity was measured using a Varioskan LUX microplate reader (Thermo Scientific, Waltham, MA, USA). For GFP detection, cells were fixed with 4% PFA after 8 h of incubation and nuclei stained with Hoechst 33342 (Thermo Scientific, Waltham, MA, USA). Images from identical fields across wells were captured using the CTL-S6 Universal M2 imaging system.

### 2.13. Biolayer Interferometry (BLI) Assay

The epitope competition relationships among mAbs were detected using Octet Red96 (ForteBio, Fremont, CA, USA). MAbs and SARS-CoV-2 wild-type (WT) RBD were diluted with BLI buffer (10 mM HEPES pH 7.4, 150 mM NaCl, 3 mM EDTA, 0.05% Tween-20). The first mAb (20 μg/mL) was loaded onto Protein A biosensors for 600 s and associated with RBD protein (2 μM) for 200 s. Subsequently, the biosensors were dipped into BLI buffer containing the second mAbs or the same first mAb (20 μg/mL) as control for 300 s. The competition binding signal was quantified by subtracting the control signal from the second mAbs’ signal. All competition binding data were normalized to the peak response (100%).

For kinetic affinity measurements, purified antibodies 3E4, 12C2, and 12D1 were immobilized on Protein A biosensors (Sartorius, Goettingen, Germany) and exposed to serially diluted SARS-CoV-2 WT RBD in BLI buffer. Biosensors were regenerated with 10 mM glycine (pH 2.0). Data were collected at 25 °C and analyzed using Octet Data Analysis software (v12.2.0.20). Sensors without antibody were used to control for background binding.

### 2.14. Negative Stain Electron Microscopy for S-Fab Complexes

Purified SARS-CoV-2 prefusion S trimer was incubated with a 1.2-fold molar excess of 12C2, 12D1, or 3E4 Fab in 20 mM Tris (pH 8.0) and 150 mM NaCl on ice for 1, 15, or 90 min. The resulting complexes were then diluted to 0.02 mg/mL, applied onto carbon-coated copper grids, blotted, and stained with 2% uranyl acetate. Grids were imaged using a Talos L120C transmission electron microscope (Thermo Fisher Scientific, Waltham, MA, USA).

### 2.15. Western Blot

Cells were lysed in NP-40 buffer (Solarbio, Beijing, China) supplemented with protease inhibitors (Solarbio, Beijing, China). Lysates were clarified by centrifugation. Samples were mixed with SDS-containing loading buffer without denaturation reagents, and equal amounts of protein were separated by SDS-PAGE and transferred onto Immobilon^®^-P PVDF membranes (Millipore, Billerica, MA, USA). Membranes were blocked with 5% non-fat milk (Sangon Biotech, Shanghai, China) in TBST (Monad, Wuhan, China), incubated with primary antibodies (3E4, 12C2 or 12D1; 10 µg/mL) for 2 h, followed by HRP-conjugated secondary antibodies. Protein bands were detected using the SuperKine™ ECL kit (Abbkine, Wuhan, China) and visualized on a ChemiDoc MP Imaging System (Bio-Rad, Hercules, CA, USA).

### 2.16. Cryo-EM Sample Preparation and Data Collection

SARS-CoV-2 spike at 0.4 mg/mL was mixed with 12C2 Fab at a 1:3.6 molar ratio and incubated for 40 s. The complex buffer contained 20 mM Tris-HCl pH 8.0, 150 mM NaCl, and 0.5% glycerol. A 3.5 μL aliquot was applied to glow-discharged holey-carbon grids (QuantiFoil R1.2/1.3), blotted for 2 s (blot force 0) under 100% humidity at 4 °C, and plunge-frozen in liquid ethane using a Vitrobot Mark IV (Thermo Fisher Scientific, Waltham, MA, USA). Grids were loaded into a CRYO ARM 300 electron microscope equipped with a Gatan K3 direct electron detector. Data were collected using SerialEM at 50,000× nominal magnification, with defocus ranging from 0.5 to 2.5 μm in super-resolution mode (pixel size 0.475 Å). Each movie stack comprised 40 frames with a total electron dose of ~40 e^−^/Å^2^.

### 2.17. Cryo-EM Data Processing

Cryo-EM data was processed with cryoSPARC v4.6.0. Recorded movies were subjected to patch motion correction and followed by contrast transfer function (CTF) estimation. For SARS-CoV-2 prefusion S trimer-12C2 Fab complex, 16,844 particles from 200 micrographs were selected and used to train a topaz model for particle picking. Using this model, particles were picked from 8650 micrographs, and 418,897 particles from good 2D classes were subjected to heterogeneous refinement, requesting four classes. The most prominent class, containing 243,370 particles, was selected for non-uniform (NU) refinement and produced a 3.02 Å resolution map.

### 2.18. Model Building and Refinement

The SARS-CoV-2 prefusion S trimer structure (PDB 7K9J) and the 12C2 Fab model predicted by AlphaFold2 were used to construct the spike-12C2 Fab complex model. Model fitting into the cryo-EM map was performed using Chimera, followed by rigid-body refinement in Coot.

## 3. Results

### 3.1. Identification of Cross-Reactive mAbs Against SARS-CoV-1 and SARS-CoV-2

To evaluate the cross-reactivity of S1-targeting antibodies elicited by infection with the ancestral SARS-CoV-2 strain, we collected blood samples in January 2021 from nine donors previously infected with the original SARS-CoV-2 strain. Using biotinylated SARS-CoV-2 S1 protein as bait, S1-specific memory B cells were isolated from peripheral blood mononuclear cells (PBMCs) by FACS. From these, 144 paired heavy- and light-chain antibody sequences were recovered through single-cell PCR and cloning.

**Figure 1:**
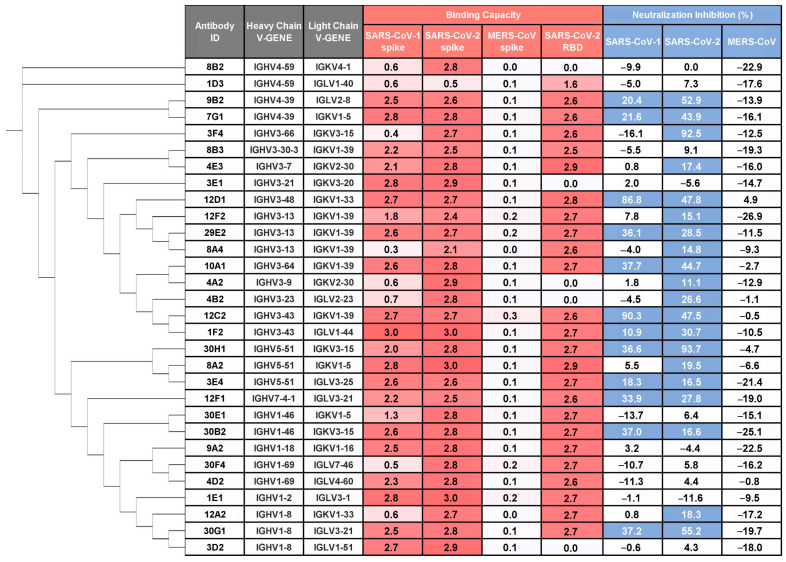
Identification of cross-reactive and cross-neutralizing mAbs against SARS-CoV-1 and SARS-CoV-2. The monoclonal antibodies (mAbs) were isolated from single B cells sorted using SARS-CoV-2 S1 protein as bait. The binding capacity (OD_450_) to prefusion-stabilized spike ectodomains of SARS-CoV-1, SARS-CoV-2, MERS-CoV and SARS-CoV-2 WT RBD is shown for mAbs in culture supernatants (5-fold dilution) and representative of two independent experiments (values are colored by a gradient of red). Pseudovirus neutralization potency (normalized to virus control = 100%) is shown for mAbs in culture supernatants (9-fold dilution). Data represent the mean from two independent replicates. Antibodies exhibiting >10% inhibition were classified as neutralizing (colored blue). Phylogenetic analysis of mAbs based on heavy chain amino acid sequences was performed using PhyloSuite.

We first assessed binding of these monoclonal antibodies (mAbs) to prefusion-stabilized S ectodomain trimers of SARS-CoV-1, SARS-CoV-2, and MERS-CoV using ELISA on culture supernatants of cells co-transfected with paired heavy and light chains (Figure S1). A high proportion (105/144; 72.9%) bound SARS-CoV-2 S ectodomain trimer, confirming that infection with the ancestral strain elicits robust S-specific responses. Among those, 30 mAbs, derived from diverse germline genes, demonstrated cross-reactivity with SARS-CoV-1 S ectodomain trimer, including 25 that targeted the RBD (Figure 1 and Figure S1). In contrast, none bound MERS-CoV S under the tested conditions (5-fold diluted supernatant), likely due to our S1-based bait strategy, given that broadly cross-reactive antibodies often target more conserved epitopes in the S2 subunit.

We next evaluated the neutralizing potential of these supernatants against vesicular stomatitis virus (VSV)-based pseudotyped virus bearing prototype SARS-CoV-2, SARS-CoV-1, or MERS-CoV spikes. Twenty mAbs exhibited neutralizing activity against SARS-CoV-2, and of these, 12 mAbs also cross-neutralized SARS-CoV-1 pseudovirus (Figure 1), underscoring their functional breadth.

### 3.2. Cross-Reactivity and Binding Capacity of Three mAbs to Sarbecovirus

**Figure 2:**
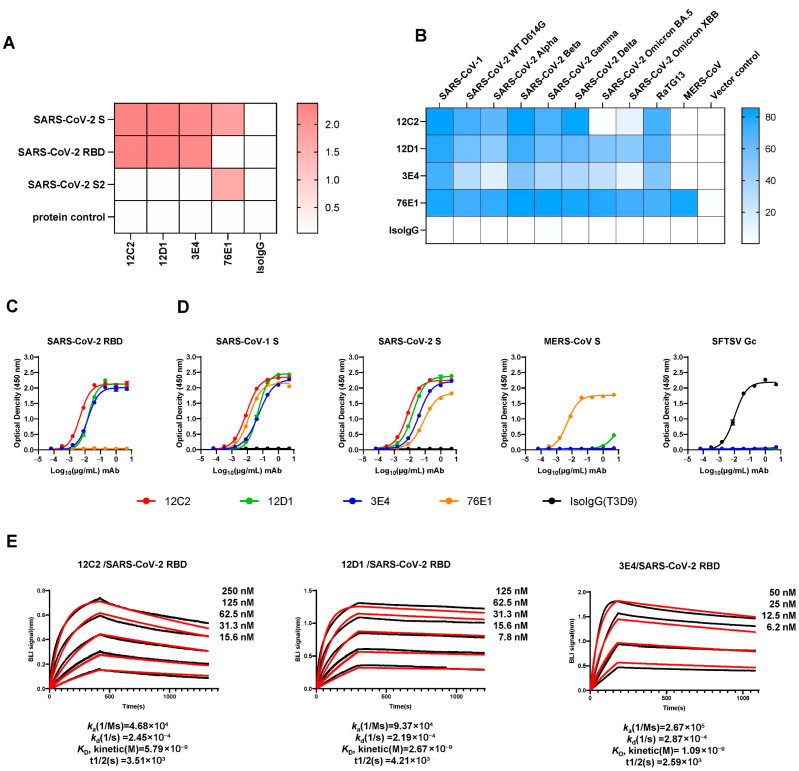
Binding profiles of the top three mAbs. (**A**) Domain-specific binding (OD_450_) of mAbs 12C2, 12D1, and 3E4 (1 µg/mL) measured by ELISA. (**B**) Flow cytometry analysis of the three RBD-targeting mAbs (1 µg/mL) binding to spike-expressing 293T cells. (**C**,**D**) Binding curves of the three mAbs to SARS-CoV-2 WT RBD (**C**) and to prefusion-stabilized spike ectodomain proteins of SARS-CoV-1, SARS-CoV-2, and MERS-CoV (**D**). The S2-targeting mAb 76E1 served as a positive control, while SFTSV Gc protein and Gc-specific mAb T3D9 (Isotype IgG, IsoIgG) were included as negative controls. Data are presented as mean ± SEM (standard error of the mean) from technical duplicates, representative of three independent experiments. (**E**) Binding affinities of mAbs to SARS-CoV-2 WT RBD measured by biolayer interferometry (BLI). Representative sensorgrams from three independent experiments are shown, with average kinetic values (*k*_a_, *k*_d_, *K*_D_ and t1/2) indicated below. The raw data are shown as black lines and the fitting curves are shown as red lines.

Following large-scale expression and revalidation, we selected three potent mAbs (12C2, 12D1 and 3E4) for further characterization. ELISA assays conducted at 1 µg/mL demonstrated that all three mAbs bind strongly to the receptor-binding domain (RBD) of the SARS-CoV-2 spike protein, without any cross-reactivity toward the S2 subunit (Figure 2A,C). This was corroborated by the specificity of the previously characterized S2-targeted antibody 76E1, which recognized only S2 and served as an effective control.

To evaluate the breadth of sarbecovirus recognition, we transfected 293T cells with plasmids encoding full-length spike proteins from SARS-CoV-1; multiple SARS-CoV-2 variants (D614G, Alpha, Beta, Gamma, Omicron BA.5, Omicron XBB); the bat coronavirus RaTG13; and MERS-CoV. Forty-eight hours post-transfection, cells were fixed and stained with each mAb (1 µg/mL), and binding was assessed via flow cytometry. The results revealed that both 12D1 and 3E4 exhibit remarkably broad reactivity: they bound effectively to SARS-CoV-1, all tested SARS-CoV-2 variants—including BA.5 and XBB—and RaTG13. In contrast, 12C2 bound most variants and SARS-CoV-1, but it failed to bind SARS-CoV-2 Omicron sublineage BA.5, indicating a relatively narrower breadth of recognition compared to 12D1. Consistent with ELISA findings, none of the mAbs bound the MERS-CoV spike (Figure 2B,D).

We next measured the binding affinities of these mAbs for the SARS-CoV-2 WT RBD using bio-layer interferometry (BLI). Antibodies were immobilized onto Protein A biosensors, and their binding signals to serially diluted RBD antigen concentrations were measured. The kinetically derived binding affinities (*K*_D_) were determined to be 5.79 nM for 12C2, 2.67 nM for 12D1, and 1.09 nM for 3E4 (Figure 2E). Complementing these findings, immunofluorescence assays confirmed the interaction of all three mAbs with the SARS-CoV-2 S, while non-reducing Western blotting validated their binding to S proteins from both SARS-CoV-1 and SARS-CoV-2 (Figure S2).

### 3.3. 12C2 Potently Neutralizes SARS-CoV-1 and Early SARS-CoV-2 Variants

**Figure 3:**
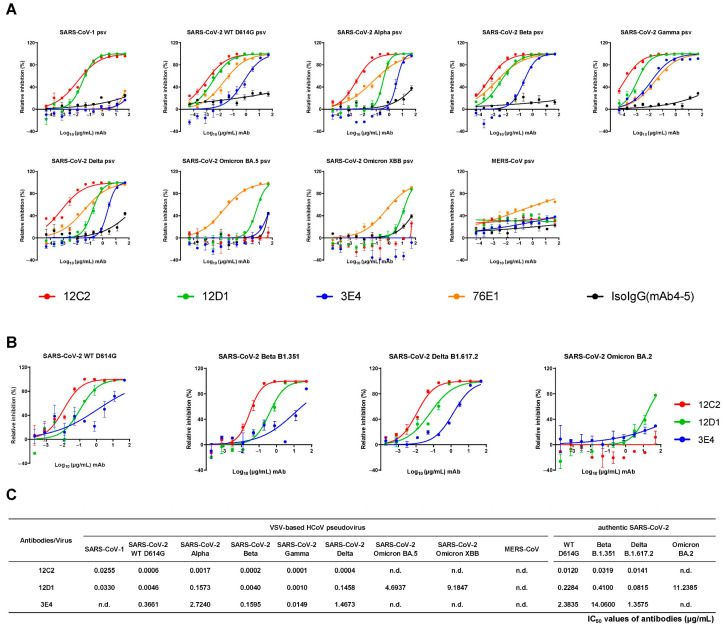
Neutralization breadth and potency of mAbs 12C2, 12D1, and 3E4 against SARS-CoV-1 and SARS-CoV-2 variants. (**A**) Neutralization activity of mAbs against SARS-CoV-1, SARS-CoV-2 (WT D614G), major SARS-CoV-2 variants (Alpha B.1.1.7, Beta B.1.351, Gamma P.1, Delta B.1.617.2, Omicron BA.5 and Omicron XBB) and MERS-CoV pseudoviruses. (**B**) Focus reduction neutralization tests (FRNT) showing the potency of mAbs against authentic SARS-CoV-2 variants (WT D614G, Beta B.1.351, Delta B.1.617.2, and Omicron BA.2). (**C**) Mean IC_50_ values derived from pseudovirus and authentic virus neutralization. n.d., not detected.

To evaluate the neutralizing potency and breadth of these three antibodies, we performed pseudovirus neutralization assays against a panel of nine VSV-based pseudoviruses, including prototype SARS-CoV-1, MERS-CoV, and seven SARS-CoV-2 subvariants (D614G, Alpha B.1.1.7, Beta B.1.351, Gamma P.1, Delta B.1.617.2, Omicron BA.5, and Omicron XBB) (Figure 3A).

12C2 exhibited potent cross-neutralization against pseudotyped SARS-CoV-1 and several SARS-CoV-2 variants (D614G, Alpha, Beta, Gamma, and Delta), with half-maximal inhibitory concentration (IC_50_) values below 0.05 μg/mL, but showed no detectable activity against Omicron BA.5 or XBB. 12D1 displayed broader neutralization breadth, with detectable activity against both Omicron BA.5 and XBB. However, its neutralizing potency against individual SARS-CoV-2 variants was around 8- to 360-fold weaker than that of 12C2. In contrast, 3E4 bound the SARS-CoV-1 spike as well as the SARS-CoV-2 spike, yet exhibited no detectable neutralization of SARS-CoV-1 (Figure 2D and Figure 3A).

For authentic SARS-CoV-2 variants (Figure 3B,C), 12D1 showed broad neutralization of all four tested strains (D614G, Beta B.1.351, Delta B.1.617.2, and Omicron BA.2). While 12C2 could not efficiently neutralize Omicron BA.2, it demonstrated the highest potency against authentic D614G (IC_50_: 0.012 μg/mL), Beta B.1.351 (IC_50_: 0.032 μg/mL), and Delta B.1.617.2 (IC_50_: 0.014 μg/mL). The mAb 3E4 exhibited moderate activity against D614G, Beta B.1.351, and Delta B.1.617.2, with IC_50_ values ranging from 1.35 to 14.06 μg/mL. Combined with binding affinity results, these results indicate that high binding affinity to recombinant protein does not necessarily predict actual neutralization potency, as seen with mAbs against severe fever with thrombocytopenia syndrome virus and Zika virus [40,41].

### 3.4. Neutralizing Mechanisms of the mAbs

**Figure 4:**
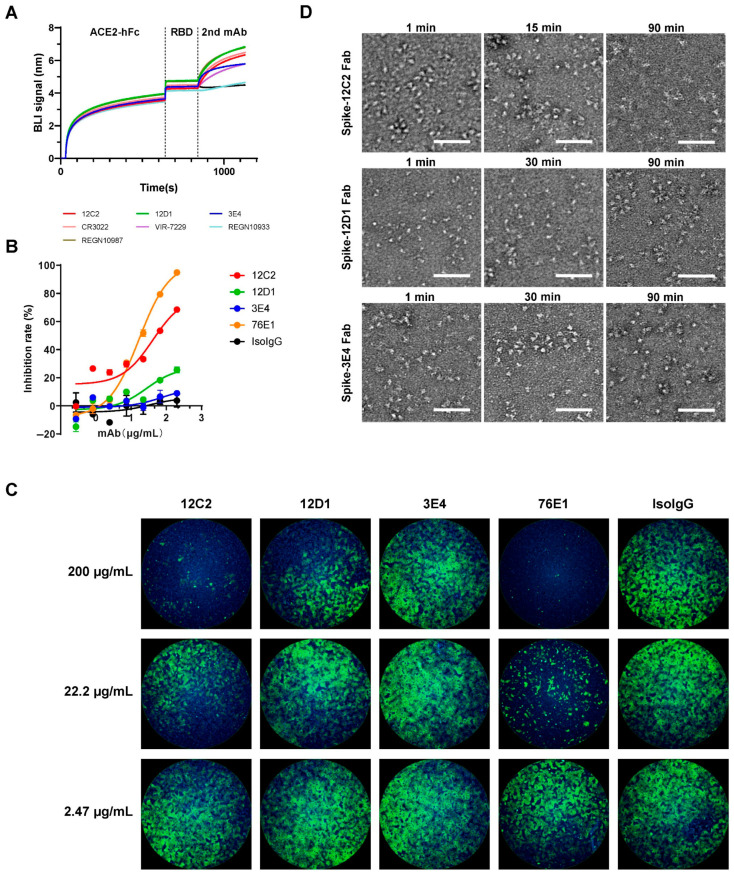
Mechanisms of neutralization by mAbs 12C2, 12D1, and 3E4. (**A**) Inhibition of hACE2 binding to recombinant SARS-CoV-2 WT RBD by mAbs, assessed via competitive BLI. hACE2-Fc was immobilized on Protein A biosensors to capture RBD, followed by measurement of mAb binding to the captured RBD. CR3022, W328-6E10, VIR-7229, REGN10933, and REGN10987 served as controls. Representative binding curves from two independent experiments are shown. (**B**,**C**) Fusion inhibition activity of the three mAbs, measured using a dual split protein (DSP) assay with 293T effector cells (co-expressing SARS-CoV-2 Spike and DSP1-7) and BHK21-hACE2 target cells (expressing DSP8-11). (**B**) Fusion inhibition curves based on luciferase activity (relative to no-antibody control, set to 100%). Data are presented as mean ± SEM from technical duplicates and are representative of three independent experiments. (**C**) GFP-based quantification of cell–cell fusion in the presence of the indicated mAbs. Fusion peptide-targeting mAb 76E1 served as a positive control. Representative images from three independent experiments are shown. (**D**) Negative-stain EM of S-Fab complexes. Purified prefusion-stabilized SARS-CoV-2 S ectodomain trimers were incubated with a 1.2-fold molar excess of 12C2, 12D1, or 3E4 Fab for 1–90 min on ice. Complexes were diluted to 0.02 mg/mL, applied to carbon-coated copper grids, and stained with 2% uranyl acetate. Images were acquired at 57,000× magnification on a Talos L120C TEM (Thermo Fisher Scientific). Scale bar: 100 nm.

To elucidate the neutralizing mechanisms of the three mAbs, we first assessed their competition with human angiotensin-converting enzyme 2 (hACE2) for binding to the SARS-CoV-2 WT RBD (Figure 4A). All three mAbs exhibited non-competitive inhibition of hACE2 binding, indicating that their epitopes reside outside the receptor-binding motif (RBM). These findings suggest that the neutralizing activity of the mAbs is mediated through mechanisms independent of direct hACE2 blockade.

Next, we evaluated the fusion-inhibitory activity of the mAbs using a spike-mediated cell–cell fusion assay with a dual-split reporter system [38,39]. This assay employs chimeric reporter proteins DSP1-7 and DSP8-11, each comprising split green fluorescent protein (spGFP) fused to split Renilla luciferase (RL). Effector cells were prepared by co-transfecting 293T cells with plasmids encoding full-length SARS-CoV-2 spike and reporter plasmid A (encoding DSP1-7). Target cells consisted of hACE2-stably expressing BHK21 cells transfected with reporter plasmid B (encoding DSP8-11). Twenty-four hours post-transfection, effector cells were dissociated into single-cell suspensions and incubated with serially diluted mAbs to enable antibody binding to spike-expressing cells. Subsequently, target cells were mixed with effector cells, and fusion activity was measured by GFP intensity and luciferase activity. The results demonstrated that 12C2 exhibited the highest fusion-inhibitory activity, followed by 12D1, while 3E4 showed negligible inhibition (Figure 4B), corroborated by qualitative GFP visualization (Figure 4C). Notably, although 12C2 exhibited more potent neutralizing activity against most SARS-CoV-2 strains compared to control mAb 76E1, 76E1 displayed more effective fusion blockade activity relative to 12C2, indicating that multiple neutralizing mechanisms may be employed by 12C2.

Furthermore, we investigated the conformational dynamics of SARS-CoV-2 S ectodomain trimers upon Fab binding using negative-stain electron microscopy at different time points after mixing: 1 min, 15 or 30 min, and 90 min (Figure 4D). Micrographs of the S-12C2 Fab complex revealed well-defined trimeric spikes with a homogeneous distribution after 1 min of incubation. In contrast, after 90 min of incubation with 12C2 Fab, recognizable spike trimers were completely lost, indicating that prolonged exposure to 12C2 Fab induces time-dependent disassembly of the S trimer. By comparison, spike treated with 12D1 or 3E4 Fab retained their trimeric architecture even after 90 min of incubation. This Fab-induced spike trimer disruption provides an additional mechanistic basis for the potent neutralization capacity of 12C2.

### 3.5. Distinct Epitope Recognition by the Three mAbs on the SARS-CoV-2 RBD

**Figure 5:**
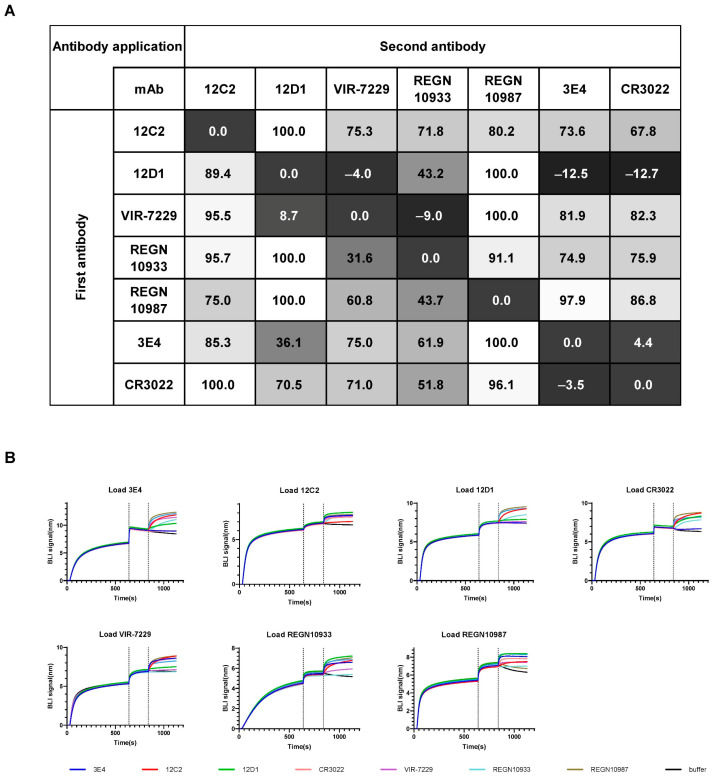
Recognition of two non-overlapping RBD epitopes by mAbs 12C2, 12D1, and 3E4. (**A**) Epitope competition assays for mAbs binding to SARS-CoV-2 WT RBD were determined by BLI. The table shows the binding signal of test antibodies expressed as a percentage of the signal from a non-competing antibody (with the highest non-competing signal set to 100%). A value < 30% was considered strong competition. Previously reported RBD cross-neutralizing mAbs (CR3022, VIR-7229, REGN10933, and REGN10987) were used as controls. Data represent the mean of two independent replicates. (**B**) Representative BLI competition binding curves from two independent experiments.

To delineate the epitopes of the three mAbs, we conducted competition-binding assays using five well-characterized cross-reactive mAbs with structurally defined epitopes on the SARS-CoV-2 RBD: RBD-2 (VIR-7229, REGN10933), RBD-5 (REGN10987), and RBD-7 (CR3022) by competition BLI. The first mAb (1st mAb) was immobilized on the sensor to capture recombinant SARS-CoV-2 WT RBD protein. The binding capacity of the second mAb (2nd mAb) to the captured RBD was determined by subtracting the binding signal of the same first antibody controls (1st mAb-RBD-1st mAb) and normalizing with the binding signal of non-competitive mAbs (set to 100%) (Figure 5A and Figure S3). Antibodies exhibiting <30% normalized signal were defined as strong competitors. Epitope competition analysis revealed that 12C2 does not compete with 12D1, 3E4, or any of the five reference mAbs, suggesting recognition of a novel antigenic site. In contrast, 12D1 exhibited strong competition with the broadly neutralizing antibody VIR-7229, while 3E4 competed with CR3022 for RBD binding. Notably, the competition between 12D1 and 3E4 was asymmetric, indicative of either overlapping epitopes or steric hindrance due to epitope proximity.

**Figure 6:**
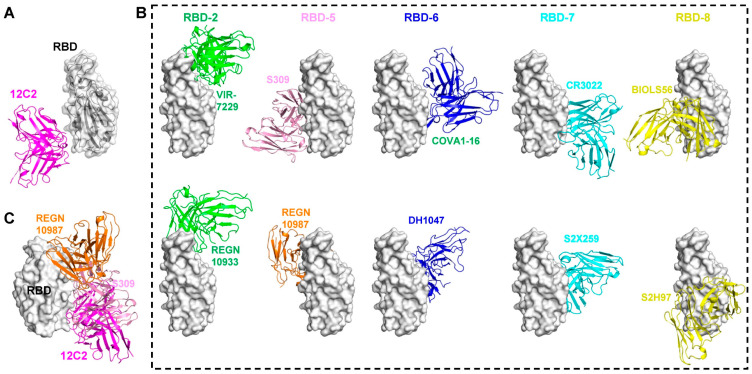
Comparison of 12C2 binding model to RBD with reported pan-sarbecovirus antibodies. (**A**) Overall structure of the 12C2 antibody bound to the SARS-CoV-2 RBD, as determined by cryo-EM reconstruction. (**B**) Comparison of representative cross-reactivity RBD-directed mAbs from the RBD-2, -5, -6, -7, and -8 communities. The RBD is depicted as a white surface, and the variable domains of the mAbs are shown as cartoon representations. RBD-2 mAb: VIR-7229 (PDB:9AU1) and REGN10933 (PDB: 6XDG), RBD-5 mAb: REGN10987 (PDB: 6XDG) and S309 (PDB: 6WS6), RBD-6 mAb: DH1047 (PDB: 7LD1) and COVA1-16 (PDB: 7JMW), RBD-7 mAb: CR3022 (PDB: 6W41) and S2X259 (PDB: 7M7W), RBD-8 mAb: BIOLS56 (PDB: 7Y3O) and S2H97 (PDB: 9ATM) were used for the comparison. (**C**) Structural alignment of the 12C2-RBD complex with REGN10987- and S309-RBD complexes.

To understand the structural basis for binding by 12C2, we determined the complex structure of SARS-CoV-2 prefusion S trimer (WT) bound to the antigen-binding fragment (Fab) of 12C2 using cryo-EM (Figure S4). Due to the preferred particle orientation and mAb-induced destabilization of the trimeric spike, obtaining a high-quality cryo-EM map of the complex was challenging. Nevertheless, the map allowed us to roughly determine the binding region of 12C2 (Figure 6A). Structural analysis showed that 12C2 recognizes the outer face of the RBD, distal to the RBM. Comparison with previously reported epitopes of cross-neutralizing mAbs targeting the RBD indicates that 12C2 overlaps significantly with the RBD-5 mAb S309, but not with other mAbs from the RBD-5 group, such as REGN10987, nor with mAbs from the RBD-2, -6, -7, and -8 groups (Figure 6B,C).

## 4. Discussion and Conclusions

As a rapidly mutating respiratory pathogen, SARS-CoV-2 continues to pose challenges for long-term vaccine efficacy [42,43]. A comprehensive understanding of the composition and mechanisms underlying protective immunity against SARS-CoV-2 is crucial for developing broadly effective countermeasures [44]. In this study, we isolated 105 mAbs targeting the S1 subunit from nine COVID-19 convalescent individuals infected with the ancestral SARS-CoV-2 strain, without prior vaccination or exposure to variant strains. Among these, 12 anti-RBD mAbs exhibited cross-reactivity with SARS-CoV-1 and demonstrated varying degrees of neutralizing activity against both SARS-CoV-1 and SARS-CoV-2. We selected three representative mAbs (12C2, 12D1, and 3E4) for detailed functional, mechanistic, and structural characterization.

Mechanistic studies revealed that none of the three mAbs inhibit viral infection by directly blocking the interaction between the recombinant RBD and the ACE2 receptor. Although further studies are needed, 12C2 appears to neutralize the virus by destabilizing the spike trimer and blocking membrane fusion, a mechanism reminiscent of S2-targeting antibodies. The modes of action of 12D1 and 3E4 remain unclear. They do not disrupt the spike trimer or interfere with the fusion process efficiently, suggesting that their antiviral activity may involve an as-yet unidentified mechanism that merit further investigation.

Epitope competition assays demonstrated that 12D1 and 3E4 recognize distinct RBD epitopes and compete with mAbs from RBD-2 and RBD-7 communities, respectively. Although 12C2 did not compete with 12D1, 3E4, or other well-characterized mAbs from the RBD-2 (VIR-7229 and REGN10933) [23,26], RBD-5 (REGN10987) [26], and RBD-7 (CR3022) [24] communities, cryo-EM structural analysis revealed that 12C2 binds to the outer face of the RBD. Its binding epitope partially overlaps with the footprint of S309, a representative RBD-5 antibody [22,45].

Previous studies have shown that the half-life-extended S309 variant (S309-LS), but not the FcγR-binding-disrupted form (S309-GRLR), protects K18-hACE2 and hFcγR transgenic mice against SARS-CoV-2 variants. Whereas another study reported that both native and Fc-silenced S309 provide comparable protection in Syrian hamsters challenged with SARS-CoV-2 [46,47]. This discrepancy may be explained by differences in the animal models used for the protective assays, and further experiments are needed to clarify these findings. However, regarding the neutralizing mechanism in vitro, S309 can interfere with attachment factor interactions on the spike and inhibit viral membrane fusion [16,22]. A similar fusion inhibition effect was observed for 12C2. Notably, we found that 12C2 also possesses the ability to destabilize and disrupt the spike trimer, a mechanism that had not been reported for S309. Given the epitope overlap between 12C2 and S309, it is plausible that S309 may also exert its antiviral activity, at least in part, through spike trimer destabilization.

S309, derived from the paired germlines IGHV1-18 and IGKV3-20, was originally isolated from a SARS-CoV-1 survivor and has been shown to neutralize both SARS-CoV-1 and SARS-CoV-2. In contrast, 12C2, which originates from the paired germlines IGHV3-43 and IGKV1-39, was isolated from an individual infected with the ancestral SARS-CoV-2 strain. The IC_50_ values of 12C2 against authentic SARS-CoV-2 WT D614G, Beta (B.1.351), and Delta (B.1.617.2) range from 12–32 ng/mL, which are around 10-fold lower than the previously reported IC_50_ values of S309-GRLR and S309-LS against SARS-CoV-2 WT D614G and B.1.351 (~200 ng/mL). Although differences in assay systems and experimental conditions may contribute to these variations, the high potency of 12C2 suggests that it, particularly in a half-life-extended format, may provide in vivo protection against SARS-CoV-2 WT D614G. Despite their different origins, S309 and 12C2 exhibit similar neutralization breadth and target overlapping epitopes on the RBD, suggesting that the epitope they recognize is both conserved and immunodominant, and can be readily elicited by natural infection in humans.

Previous studies have suggested that potent cross-neutralizing antibodies against both SARS-CoV-1 and SARS-CoV-2 are rare in individuals infected with the ancestral SARS-CoV-2 strain [25]. However, in our study, approximately 12 out of 105 S-binding mAbs displayed cross-neutralizing activity, indicating that such antibodies may be more common than previously reported. This discrepancy in frequency is likely due to differences in antibody discovery approaches, and further research is warranted to better define the prevalence and induction of broadly reactive antibodies in the human immune response. Overall, our findings provide important insights into antibody-mediated viral inhibition and highlight the potential of targeting conserved, readily inducible epitopes for broad sarbecovirus neutralization and vaccine design.

## Data Availability

All relevant data are within the manuscript. Further inquiries can be directed to the corresponding author.

## Supplementary Materials

The following supporting information can be downloaded at: https://www.mdpi.com/article/10.3390/v17101285/s1, Figure S1: Recognition of cross-reactive antibodies; Figure S2: Antibody validation by Immunofluorescence and Western Blotting; Figure S3: Epitope competition assay; Figure S4: Cryo-EM reconstruction of SARS-CoV-2 prefusion S trimer complexed with 12C2 Fab.

## Abbreviations

The following abbreviations are used in this manuscript:

SARS-CoV-1	Severe acute respiratory syndrome coronavirus
SARS-CoV-2	Severe acute respiratory syndrome coronavirus 2
COVID-19	Coronavirus disease 2019
S	spike
RBD	Receptor binding domain
NTD	N-terminal domain
PBMC	Peripheral blood mononuclear cell
mAb	Monoclonal antibodies
ELISA	Enzyme-linked immunosorbent assay
PFA	Paraformaldehyde
BLI	Bio-layer interferometry
*K*_D_	Kinetically derived binding affinity
IC_50_	Half-maximal inhibitory concentration
hACE2	Human angiotensin-converting enzyme 2
RBM	Receptor-binding motif
spGFP	Split green fluorescent protein
RL	Renilla luciferase
ABSL-3	Animal Biosafety Level-III Laboratory
PEI	Polyethyleneimine
DMEM	Dulbecco’s Modified Eagle’s Medium
FBS	Fetal bovine serum
Cryo-EM	Cryo-electron microscopy
CTF	Contrast transfer function
Fab	Antigen-binding fragment
scFv	Single-chain variable fragment
SEM	Standard error of the mean
FRNT	Focus reduction neutralization test
MB	Memory B
FSC	Fourier shell correlation
