# Supplementary material for: Broadly Sarbecovirus-Neutralizing Antibodies Induced by Ancestral SARS-CoV-2 Infection
Source: Viruses. 2025 Sep 23;17(10):1285. doi: 10.3390/v17101285 (PMC12567802; doi:10.3390/v17101285)
Supplement: Supplementary file 1 [file viruses-17-01285-s001.zip › viruses-3859047-supplementary.pdf]

Supplementary Materials

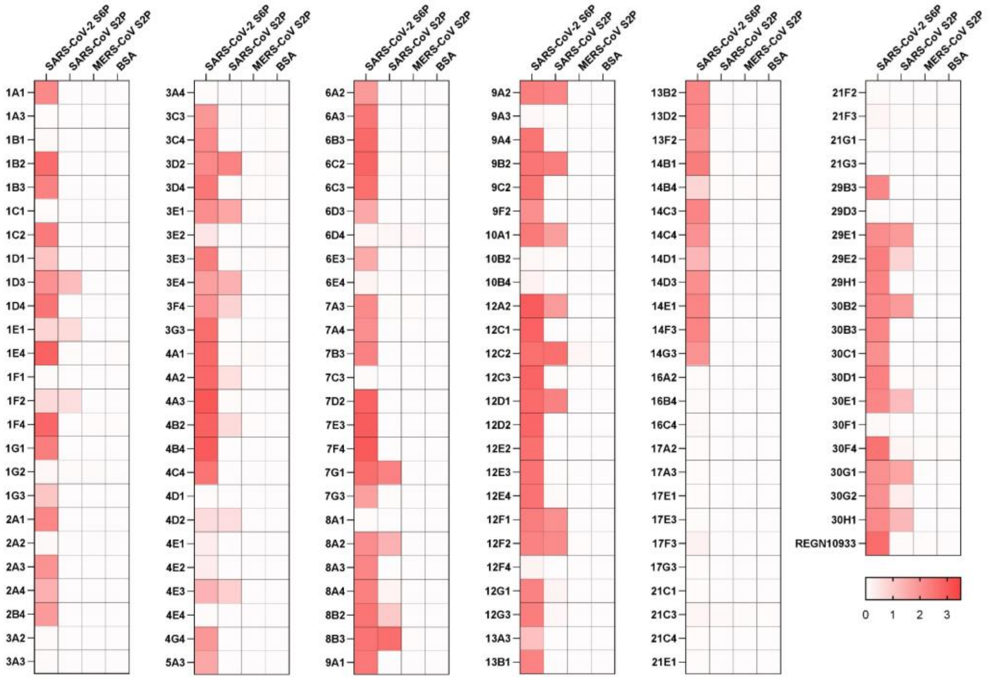

Figure S1. Recognition of cross-reactive antibodies. Paired heavy and light chains of 144 mAbs derived from S1-specific memory B (MB) cells were transfected into 293T cells. Antibody-containing supernatants were collected 48 hours post-transfection and screened by ELISA for binding to coronavirus spike proteins. Data represent the binding signals of 5-fold diluted supernatants to the spike proteins of SARS-CoV-1, SARS-CoV-2, and MERS-CoV. BSA served as a negative protein control, and the RBD-targeting mAb REGN10933 was included as a positive control.

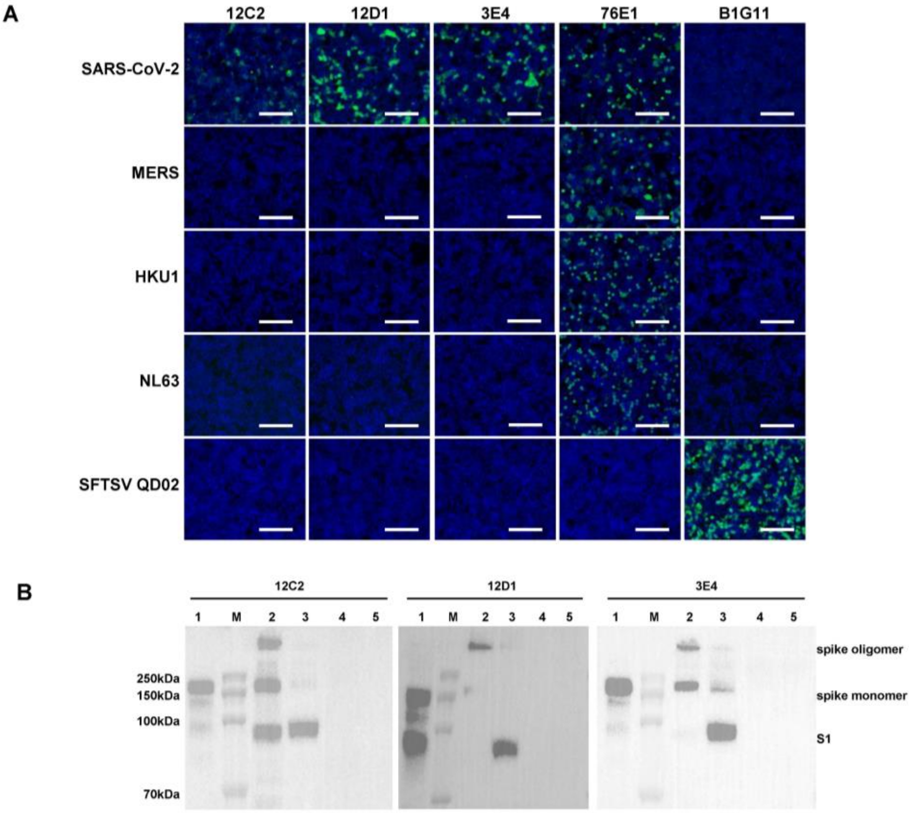

Figure S2. Antibody validation by Immunofluorescence and Western Blotting. (**A**) Immunofluorescence assay of mAb binding. 293T cells transfected with full-length coronavirus spike plasmids were fixed 48 hours post-transfection and stained with 12C2, 12D1 or 3E4 (10 μg/mL). The broadly neutralizing coronavirus mAb 76E1 and the SFTSV Gn-specific mAb B1G11 served as controls. Scale bar: 100 μm. (**B**) Western blot analysis of mAb binding. Lysates from 293T cells transfected with full-length coronavirus spike plasmids were probed with the three mAbs under non-reducing conditions. Lanes: 1: SARS-CoV-2 prefusion-stabilized spike ectodomain protein; 2: SARS-CoV-1 full-length spike-transfected cell lysate; 3: SARS-CoV-2 full-length spike-transfected cell lysate; 4: MERS-CoV full-length spike-transfected cell lysate; 5: Empty vector-transfected cell lysate (negative control). Representative images from two (A) or three (B) independent experiments are shown.

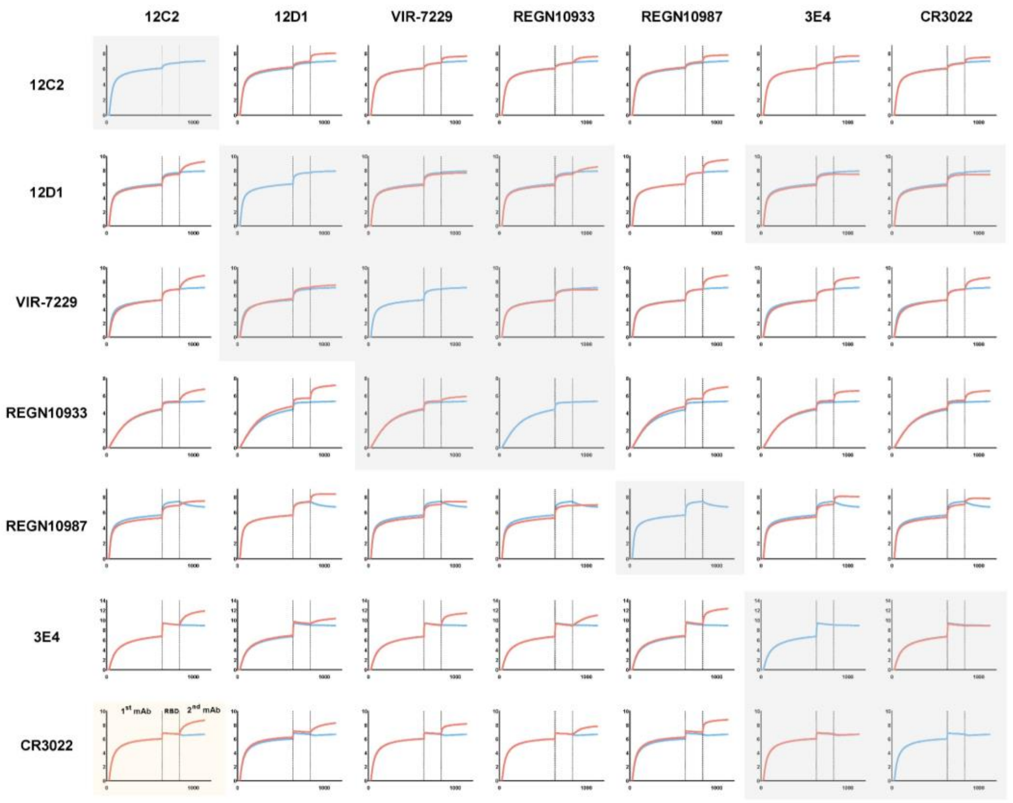

Figure S3. Epitope competition assay. Epitope competition among mAbs was assessed using BLI. The first antibody (1st mAb) was immobilized on Protein A biosensors to capture SARS-CoV-2 WT RBD, followed by evaluation of the binding of the second antibody (2nd mAb). The matrix graph displays the same first mAbs (rows, left-axis labels) and secondary mAbs (columns, top-axis labels). Diagonal plots (1st mAb-RBD-1st mAb, blue sensorgrams) served as negative controls to confirm occupation of Fc-binding sites on the Protein A biosensors. A substantial increase in BLI signal upon addition of the 2nd mAb indicates non-competing epitopes, whereas little or no increase suggests competition for the overlapped RBD epitope. Representative curves from two independent experiments are shown.

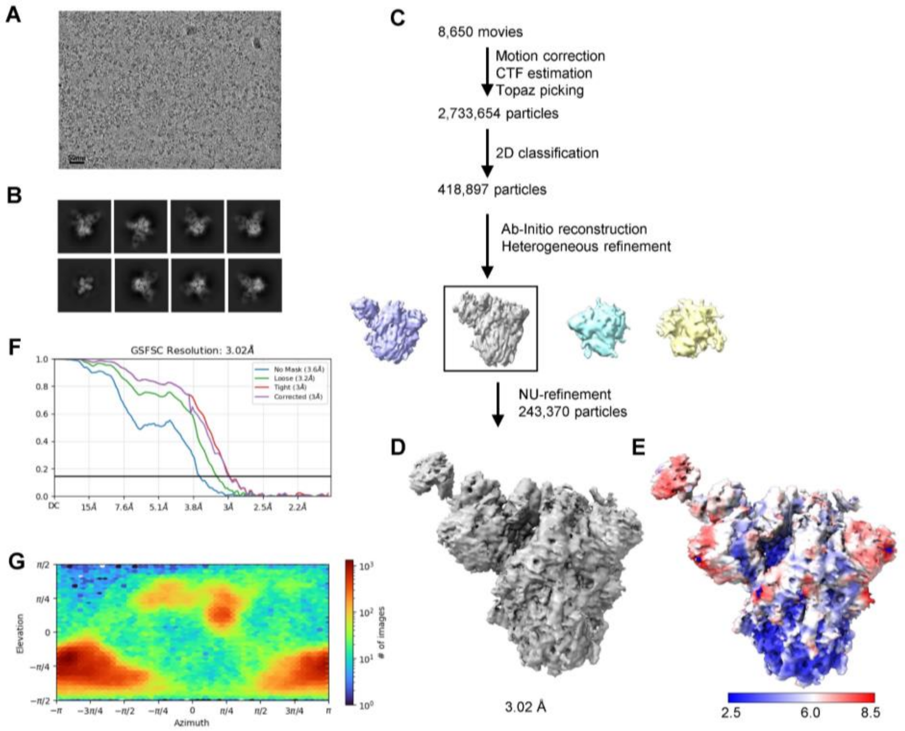

Figure S4. Cryo-EM reconstruction of SARS-CoV-2 prefusion S trimer complexed with 12C2 Fab. (**A**) Representative cryo-EM image of SARS-CoV-2 prefusion S trimer-12C2 Fab complex. (**B**) 2D class averages of SARS-CoV-2 prefusion S trimer-12C2 Fab complex. (**C**) Flowchart of cryo-EM data processing. (**D**) Cryo-EM density map colored by local resolution. **(E)** Fourier shell correlation (FSC) curve was calculated using two independent half maps, and resolution was estimated using the FSC=0.143 cutoff. (**F**) Euler distribution of the refined particles.
